# Supplementary material for: Preparing for the future: A mixed methods study protocol on AI awareness and educational integration in Qatar’s Primary Health Care Workforce
Source: PLoS One. 2026 Sep 15;21(9):e0345347. doi: 10.1371/journal.pone.0345347 (PMC13577504; doi:10.1371/journal.pone.0345347)
Supplement: Supplementary file 1 [file pone.0345347.s001.pdf]

Integrating AI awareness and education among healthcare professionals in PHCC

Preparing for the Future: A Mixed Methods Study on AI Awareness and Educational Integration in Qatar's Primary Health Care Workforce"

* Required

Study background and consent to participate in the study

Study background This survey aims to investigate the integration of Artificial Intelligence (AI) education within primary care settings in Qatar. It focuses on clinicians' readiness, awareness, and perception of AI tools and their potential impact on improving clinical outcomes and operational efficiency. Your responses will help identify educational needs and recommendations for enhancing AI education in primary care settings within PHCC. This communication is to invite you to participate in Primary Health Care Corporation (PHCC) Institutional Review Board (IRB) approved research study with Ref No: BUHOOTH-00096). Further details about the study are provided below.

Consent Form

You are invited to participate in a research study titled "**I**ntegrating Artificial Intelligence (AI) Education into Primary Health Care Settings in Qatar: A Mixed-Methods Study" being conducted by Dr Muslim Abbas Syed from Clinical Research Department. The conduct of this study will adhere to the PHCC IRB approved protocol.

The IRB approved approach to invite individuals for the study is by sending a email with a link to an online questionnaire. You can access the survey by clicking the "I agree/ Continue/ Next" button at the end of this introductory page. Before agreeing to participate in the survey, please note the following:

· Your participation is completely voluntary.

· We appreciate your voluntary contribution to science by participating in this research. However, if you are employed by PHCC, this is not an official work task.

The questionnaire includes various components which mainly include structured questions pertaining to awareness and knowledge about application of AI tools in primary care services, training and educational needs, perceptions and attitudes towards AI, infrastructure readiness, impact on clinical decision making and patient care and recommendations for enhancing AI. Completing the questionnaire will approximately take 15-20 Minutes minutes.

· Your responses will be completely anonymized. The research team will not have access to your personal details.

· The data collected will remain confidential and anonymous. Records will be monitored and may be audited by the IRB while assuring confidentiality.

· The anonymized results of the study will be presented or published in various media such as oral presentations, media articles, research paper.

· You will not receive payment for participation in this study.

· There are no risks with participating in this study.

· Refusal to participate or withdrawal from the study will involve no penalty or loss of benefits to which the subject is otherwise entitled, and neither will it affect their relationship with PHCC or any other organizations.

1. Since your participation in the survey is anonymous, you can ***NOT*** recall (delete) your responses once submitted

◯ Continue

◯ Discard

2.

Who should you contact if you have any questions about the study?

If you have any questions about the study, please contact Dr Muslim Abbas Syed at 71234476 or at musyed@phcc.gov.qa

Who should you contact if you have any concerns or questions about your rights?

If you have concerns about the study or questions about your rights as a research participant, you can contact the PHCC Department of Clinical Research using the below contact details:

· Office phone (7 am -2 pm Sunday- Thursdays excluding public holidays) : 40270971 or 40271102 or 40271030

· Email: researchsection@phcc.gov.qa

Please note that by clicking the button below (I agree/ I disagree), you are ***voluntarily consenting*** to participate in the study.

◯ I agree

◯ I disagree

Demographic details

3. From which Primary Health Care Center are you participating in this online survey? *

- ◯ Rawdat Al Khail
- ◯ Al Wakra
- ◯ Omar Bin Al Khattab
- ◯ West Bay
- ◯ Al Thumama
- ◯ Al Sadd
- ◯ Airport
- ◯ Al Mashaf
- ◯ Umm Ghuwailina
- ◯ South Wakra
- ◯ Umm Slal
- ◯ Leabaib
- ◯ Gharrrafat Al Rayyan
- ◯ Qatar University
- ◯ Al Khor
- ◯ Madinat Khalifa
- ◯ Al Daayen
- ◯ Al Ruwais
- ◯ Al Kaaban
- ◯ Muaither

- ◯ Al Wajbah
- ◯ Abu Baker Al Siddiq
- ◯ Mesaimeer
- ◯ Al Rayyan
- ◯ Al Waab
- ◯ Abu Nakhla
- ◯ Sheehaniya
- ◯ Umm Al Seneem
- ◯ Al Karaana
- ◯ PHCC Headquarters

4. Gender? *

- ◯ Male
- ◯ Female

5. Age in years?

6. What is your occupation ? *

- ◯ Physicians
- ◯ Dentists
- ◯ Pharmacist
- ◯ Nurses
- ◯ Laboratory Tech
- ◯ Radiology Tech
- ◯ Dietitian
- ◯ Health Educator
- ◯ Physiotherapist
- ◯ Administrative staff
- ◯ Other

Awareness and knowledge about AI applications in health care

7. How familiar are you with application of AI technologies in health care (particularly in delivery of primary care services)?

- ◯ Not familiar
- ◯ Somewhat familiar
- ◯ Familiar
- ◯ Very familiar

8. How did you learn about AI in healthcare and potential of its utilization in primary care services?

- ☐ Formal training
- ☐ Self-learning
- ☐ Conferences
- ☐ Other

9. Please comment on the following list of AI tools with the potential of being utilized in primary care settings

	I don't know about it	I'm aware about it	I have used it
Qatar Biobank & Genomics	◯	◯	◯
Malaffi & Cerner Integration	◯	◯	◯
AI-powered triage systems (Tools like Babylon Health or Infermedica)	◯	◯	◯
Radiology AI	◯	◯	◯
Dermatology AI (Tools like SkinVision or VisualDx)	◯	◯	◯
Risk stratification models	◯	◯	◯
Natural Language Processing (NLP)	◯	◯	◯
Chatbots for appointment scheduling and FAQs (examples Microsoft Health Bot, Ada Health)	◯	◯	◯
Smart queue management	◯	◯	◯
AI for lifestyle coaching and chronic disease management (Tools like Lark Health or Omada)	◯	◯	◯

	I don't know about it	I'm aware about it	I have used it
Wearable integration and remote monitoring (e.g., Fitbit, Apple Watch)	◯	◯	◯
AI for cybersecurity (Tools like Darktrace or Cognito	◯	◯	◯

10. Awareness about the ethical implications of using AI in healthcare

	Strongly Agree	Agree	Neutral	Disagree	Strongly Disagree
I am aware of ethical considerations related to AI in healthcare	◯	◯	◯	◯	◯
I understand the importance of patient data privacy in AI systems	◯	◯	◯	◯	◯
I believe AI should be used responsibly to avoid bias and harm	◯	◯	◯	◯	◯
I'm aware about guidelines, rules and regulations governing responsible AI use	◯	◯	◯	◯	◯

Training and educational needs

11. Do you feel adequately trained to use AI tools in your clinical practice?

- ◯ Yes – I've received formal training
- ◯ Somewhat – I've had informal exposure
- ◯ No – I need more training
- ◯ I don't think training is necessary

12. What type of AI training would be most beneficial for your role?

- ◯ Hands-on workshops on AI tools used in PHCC
- ◯ Online courses on AI in healthcare
- ◯ Case-based learning on AI-supported decision-making
- ◯ I'm not sure what training would help

13. In your opinion please select the core AI competencies that you think are required from healthcare professionals providing primary care services

	Strongly agree	Agree	Neutral	Disagree	Strongly disagree
**Foundational Knowledge** – Understanding AI concepts and healthcare applications.	◯	◯	◯	◯	◯
**Critical Appraisal** – Evaluating AI tools for accuracy, fairness, and relevance.	◯	◯	◯	◯	◯
**Medical Decision-Making** – Knowing when and how to use AI in clinical care.	◯	◯	◯	◯	◯
**Technical Use** – Operating AI tools effectively and troubleshooting.	◯	◯	◯	◯	◯
**Patient Communication** – Explaining AI use and addressing concerns.	◯	◯	◯	◯	◯
**Awareness of Unintended Consequences** – Recognizing biases, errors, and ethical risks.	◯	◯	◯	◯	◯

14. What type of AI training in your opinion will be most beneficial for healthcare professionals working in primary care services (please select all options that you think are applicable in your opinion)

- ☐ Introductory AI modules integrated into medical curricula
- ☐ AI-enhanced simulation training at undergraduate and postgraduate level
- ☐ CPD Courses: Fundamentals of AI in Healthcare
- ☐ Evidence-based CPD relating to AI in healthcare aligned with PHCC's strategic goals and national standards
- ☐ AI-focused workshops and seminars for clinical staff

Perceptions and attitudes towards AI

15. In your opinion, what is the level of skills (AI expertise) required by healthcare professionals working in primary care settings?

- ◯ Basic skills (Practical ability to use clinical AI tools)
- ◯ Proficient skills (Ability to critically assess the utility of clinical AI applications and their outputs as well as the ethical implications of integrating AI in healthcare (in addition to basic skills)
- ◯ Expert skills (Deep technical understanding of machine learning combined with clinical expertise that enables experts to drive meaningful advancements in medical AI (in addition to proficient skills)

16. Please indicate your level of agreement with the following statements

	Strongly agree	Agree	Neutral	Disagree	Strongly disagree
AI can improve clinical outcomes	◯	◯	◯	◯	◯
AI tools are trustworthy	◯	◯	◯	◯	◯
I am concerned about the use of AI in patient care.	◯	◯	◯	◯	◯

Impact of AI on clinical decision making and patient care

17. In your opinion, how could AI tools most positively influence clinical decision-making in primary care? (Select all that are applicable in your opinion)

	Strongly agree	Agree	Neutral	Disagree	Strongly disagree
Improve diagnostic accuracy	◯	◯	◯	◯	◯
Reduce time spent on documentation	◯	◯	◯	◯	◯
Enhance patient risk stratification	◯	◯	◯	◯	◯
Will improve patient centered care	◯	◯	◯	◯	◯

18. What concerns do you have about using AI in clinical decision-making?

	Strongly agree	Agree	Neutral	Disagree	Strongly disagree
Risk of bias or errors in AI recommendations	◯	◯	◯	◯	◯
Lack of transparency in AI algorithms	◯	◯	◯	◯	◯
Reduced clinician autonomy	◯	◯	◯	◯	◯
Patient trust and acceptance of AI	◯	◯	◯	◯	◯

Implementation & Integration

19. Does your health center have the necessary infrastructure to support AI tools?

- ○ Yes
- ○ No
- ○ Not sure

20.

	Very likely	Somewhat likely	Neither likely nor unlikely	Somewhat unlikely	Very unlikely
How likely are you to use AI tools if they are integrated into your clinical workflow (e.g., EMR, triage systems)?	○	○	○	○	○

21. What challenges exist in implementing AI in your primary health care center? (Briefly describe or list the challenges that exist in your opinion)

22. What do you think is the biggest barrier to implementing AI in PHCC clinical settings?

	Strongly agree	Agree	Neutral	Disagree	Strongly disagree
Lack of training and awareness	◯	◯	◯	◯	◯
Limited infrastructure or technical support	◯	◯	◯	◯	◯
Ethical and legal concerns	◯	◯	◯	◯	◯
Resistance from healthcare professionals	◯	◯	◯	◯	◯

23. What recommendations do you have for improving AI education for clinicians?

24. The researcher intends to explore this AI topic further in a personal in-depth interview and Focus Group Discussion. Would you be interested in participating in that part of the project, too? *

◯ Yes

◯ Maybe

◯ No

25. Please provide your name to help us get in contact

26. Please provide your email to help us get in contact

27. Please provide your phone number to help us get in contact (optional)

This content is neither created nor endorsed by Microsoft. The data you submit will be sent to the form owner.

Microsoft Forms
