# Supplementary material for: Preparing for the future: A mixed methods study protocol on AI awareness and educational integration in Qatar’s Primary Health Care Workforce
Source: PLoS One. 2026 Sep 15;21(9):e0345347. doi: 10.1371/journal.pone.0345347 (PMC13577504; doi:10.1371/journal.pone.0345347)
Supplement: Supplementary file 2 [file pone.0345347.s002.docx]

**Preparing for the Future: A Mixed Methods Study on AI Awareness and Educational Integration in Qatar’s Primary Health Care Workforce**

Dear Colleague

Greetings

Thank you for accepting to review and evaluate the study questionnaire tool. This tool was created aiming to explore healthcare professionals’ current understanding of AI, their attitudes toward its use in healthcare, and the systemic barriers to AI education and implementation. The inclusion of all healthcare professionals within PHCC ensures a holistic view across disciplines and roles. The study will assess the awareness, perceptions, and educational needs related to Artificial Intelligence (AI) healthcare related applications among clinical professionals in Qatar’s Primary Health Care Corporation. The ultimate goal is to evaluate the readiness of primary care infrastructure for integrating AI education.

Please read each test item in the questionnaire form. For each item, please indicate whether it is essential, useful but not essential, or not necessary as a contributor to the overall measured construct. Please ensure to use the appropriate code instead of writing text for the first two columns (rating and reason).

**Note: You can decline the task if you feel that you don’t have enough experience with AI tools in healthcare.**

# I. Demographic details

| **Instrument Q#** | **Panelist rating**  **1=not necessary**  **2=useful but not essential**  **3=Essential** | **Reason for unfavorable (not essential) rating**  **1=irrelevant to the construct tested**  **2=Too technical and difficult for the audience**  **3=Repeated elsewhere**  **4=others (specify)e** | **Any comments or suggestions to improve the phrasing** |
| --- | --- | --- | --- |
| **Q3** |  |  |  |
| **Q4** |  |  |  |
| **Q5** |  |  |  |
| **Q6** |  |  |  |

# II. Awareness and knowledge about AI applications in health care

|  | **Panelist rating**  **1=not necessary**  **2=useful but not essential**  **3=Essential** | **Reason for unfavorable (not essential) rating**  **1=irrelevant to the construct tested**  **2=Too technical and difficult for the audience**  **3=Repeated elsewhere**  **4=others (specify)e** | **Any suggestions to improve the phrasing** |
| --- | --- | --- | --- |
| **Q7** |  |  |  |
| **Q8** |  |  |  |
| **Q9** |  |  |  |
| **Q10** |  |  |  |

# III. Training and educational needs

|  | **Panelist rating**  **1=not necessary**  **2=useful but not essential**  **3=Essential** | **Reason for unfavorable (not essential) rating**  **1=irrelevant to the construct tested**  **2=Too technical and difficult for the audience**  **3=Repeated elsewhere**  **4=others (specify)e** | **Any suggestions to improve the phrasing** |
| --- | --- | --- | --- |
| **Q11** |  |  |  |
| **Q12** |  |  |  |
| **Q13** |  |  |  |
| **Q14** |  |  |  |
| **Q15** |  |  |  |
| **Q16** |  |  |  |

# IV. Impact of AI on clinical decision making and patient care

|  | **Panelist rating**  **1=not necessary**  **2=useful but not essential**  **3=Essential** | **Reason for unfavorable (not essential) rating**  **1=irrelevant to the construct tested**  **2=Too technical and difficult for the audience**  **3=Repeated elsewhere**  **4=others (specify)e** | **Any suggestions to improve the phrasing** |
| --- | --- | --- | --- |
| **Q17** |  |  |  |
| **Q18** |  |  |  |

# V. Implementation & Integration

|  | **Panelist rating**  **1=not necessary**  **2=useful but not essential**  **3=Essential** | **Reason for unfavorable (not essential) rating**  **1=irrelevant to the construct tested**  **2=Too technical and difficult for the audience**  **3=Repeated elsewhere**  **4=others (specify)e** | **Any suggestions to improve the phrasing** |
| --- | --- | --- | --- |
| **Q19** |  |  |  |
| **Q20** |  |  |  |
| **Q21** |  |  |  |
| **Q22** |  |  |  |
| **Q23** |  |  |  |
