# Supplementary material for: Preparing for the future: A mixed methods study protocol on AI awareness and educational integration in Qatar’s Primary Health Care Workforce
Source: PLoS One. 2026 Sep 15;21(9):e0345347. doi: 10.1371/journal.pone.0345347 (PMC13577504; doi:10.1371/journal.pone.0345347)
Supplement: Supplementary file 3 [file pone.0345347.s003.docx]

**Revision Log (Iterative Amendments)**

| **Date** | **Instrument Q#** | **Issue identified** | **Change made** | **Reason/Justification** | **Reviewer ID** | **Version** |
| --- | --- | --- | --- | --- | --- | --- |
|  |  |  |  |  |  |  |
|  |  |  |  |  |  |  |
|  |  |  |  |  |  |  |
|  |  |  |  |  |  |  |
|  |  |  |  |  |  |  |
|  |  |  |  |  |  |  |
|  |  |  |  |  |  |  |
|  |  |  |  |  |  |  |
|  |  |  |  |  |  |  |
|  |  |  |  |  |  |  |
