# Supplementary material for: Preparing for the future: A mixed methods study protocol on AI awareness and educational integration in Qatar’s Primary Health Care Workforce
Source: PLoS One. 2026 Sep 15;21(9):e0345347. doi: 10.1371/journal.pone.0345347 (PMC13577504; doi:10.1371/journal.pone.0345347)
Supplement: Supplementary file 4 [file pone.0345347.s004.docx]

**Interview Guide: Preparing for the Future: A Mixed Methods Study on AI Awareness and Educational Integration in Qatar’s Primary Health Care Workforce**

**Introduction**

The rapid advancement of Artificial Intelligence (AI) technologies has created new opportunities for improving clinical decision-making, operational efficiency, and patient outcomes in primary health care. However, the successful integration of AI tools depends on clinicians' awareness, readiness, and education. This research aims to assess the current state of AI knowledge among clinicians in Qatar, identify educational gaps, and propose strategies for effective AI education integration. In this interview, we will discuss the integration of AI education within primary care settings in Qatar, focusing on healthcare professionals' readiness, awareness, and perception of AI tools and their potential impact on improving clinical outcomes and operational efficiency. I want to assure you that your responses will be confidential and will only be used for research purposes. Your personal information will be kept private, and participation in this interview is entirely voluntary. At any point you feel uncomfortable or decide to discontinue the interview just let me know and we will stop.

**Section 1: Awareness and Knowledge of AI**

1. Can you describe your current understanding of Artificial Intelligence (AI) in healthcare?
2. Have you encountered or used any AI tools in your clinical practice? If yes, which ones?
3. Where did you learn about AI in healthcare (e.g., formal training, self-learning, conferences)?
4. How confident do you feel in your knowledge of AI technologies relevant to primary care?

**Section 2: Training and Educational Needs**

5. Have you received any formal training or education on AI in your medical career?
6. What kind of training or resources would you find helpful to better understand and use AI in your practice?
7. Are there any specific areas of AI (e.g., diagnostics, predictive analytics, patient triage) you would like to learn more about?
8. What barriers do you face in accessing AI-related education or training?

**Section 3: Perceptions and Attitudes**

9. What is your general attitude toward the use of AI in primary care?
10. What benefits do you think AI could bring to your clinical practice?
11. Do you have any concerns or reservations about using AI tools in patient care?
12. How do you think patients might perceive the use of AI in their care?

**Section 4: Infrastructure and Readiness**

13. Do you think your health center has the necessary infrastructure (e.g., IT systems, internet access, technical support) to support AI tools?
14. Are there any organizational or technical challenges that might hinder the integration of AI in your setting?
15. How prepared do you think your team or institution is for adopting AI technologies?

**Section 5: Impact on Clinical Decision-Making and Patient Care**

16. In what ways do you think AI could support or enhance clinical decision-making?
17. Can you think of any scenarios where AI could improve patient outcomes or operational efficiency?
18. Do you believe AI could change the way you interact with patients or manage care? If so, how?

**Section 6: Recommendations for AI Education**

19. What recommendations would you make for improving AI education for clinicians in primary care?
20. How should AI training be delivered (e.g., workshops, online modules, integrated into CME)?
21. What role should healthcare institutions or policymakers play in supporting AI education?

**Conclusion:**

Thank you for participating in the interview and providing invaluable feedback. In this interview, we discussed the integration of AI education within primary care settings in Qatar, focusing on clinicians' readiness, awareness, and perception of AI tools and their potential impact on improving clinical outcomes and operational efficiency. Please do let me know if there are areas that have not been discussed and need further discussion or explanation.
