# Supplementary material for: Preparing for the future: A mixed methods study protocol on AI awareness and educational integration in Qatar’s Primary Health Care Workforce
Source: PLoS One. 2026 Sep 15;21(9):e0345347. doi: 10.1371/journal.pone.0345347 (PMC13577504; doi:10.1371/journal.pone.0345347)
Supplement: Supplementary file 5 [file pone.0345347.s005.docx]

**Focus Group Guide: Preparing for the Future: A Mixed Methods Study on AI Awareness and Educational Integration in Qatar’s Primary Health Care Workforce**

**Introduction**

**Introduction**

Thank you for participating in this FGD. The purpose of this discussion is to assess the current state of AI knowledge among clinicians in Qatar, identify educational gaps, and propose strategies for effective AI education integration.

Importance of Study:

The rapid advancement of Artificial Intelligence (AI) technologies has created new opportunities for improving clinical decision-making, operational efficiency, and patient outcomes in primary health care. However, the successful integration of AI tools depends on clinicians' awareness, readiness, and education. In this Focus Group Discussion, we will discuss the integration of AI education within primary care settings in Qatar, focusing on clinicians' readiness, awareness, and perception of AI tools and their potential impact on improving clinical outcomes and operational efficiency

Confidentiality and Consent:

I want to assure you that your responses will be confidential and will only be used for research purposes. Your personal information will be kept private, and participation in this FGD is entirely voluntary.

Overview of Topics to be Discussed:

During our conversation, we will cover several themes, including your understanding of AI awareness and knowledge, training and educational needs relating to AI, perceptions and attitudes towards AI in healthcare, infrastructure readiness and impact of AI of clinical practice within primary care settings within PHCC.

Duration of the Interview:

The FGD will take approximately 1 hour and 15 minutes.

Opportunity for Questions:

Before we start, do you have any questions about the study or the FGD process?

Main themes to be discussed in the FGD:

**Theme: Assess Current Awareness and Knowledge of AI among healthcare professionals**

What do you understand by Artificial Intelligence in healthcare?

Have you encountered or used any AI tools in your clinical practice?

How would you rate your current knowledge of AI technologies?

**Theme: Examine Training and Educational Needs**

Have you received any formal training on AI in healthcare?

What kind of educational resources or training would help you better understand AI?

What challenges do you face in accessing AI-related training?

**Theme: Explore Perceptions and Attitudes towards AI in Healthcare**

What are your thoughts on the use of AI in primary care?

Do you trust AI tools to assist in clinical decision-making?

What benefits or concerns do you associate with AI in healthcare?

**Theme: Evaluate the Readiness of Primary Care Infrastructure**

Do you think your health center is equipped to integrate AI technologies?

What infrastructural or resource limitations exist in your center?

What support would be needed to implement AI tools effectively?

**Theme: Determine the Impact of AI Education on Clinical Decision-Making and Patient Care**

How do you think AI education could influence your clinical decisions?

Can AI tools improve patient care and operational efficiency?

What examples can you share where AI made a difference in care delivery?

**Theme: Propose Recommendations for Enhancing AI Education in Primary Care**

What recommendations would you make to improve AI education for clinicians?

How should AI training be integrated into existing professional development programs?

What role should policymakers and institutions play in promoting AI education?

**Conclusion**

- We covered important themes in this FGD during our conversation including your understanding of AI awareness and knowledge, training and educational needs relating to AI, perceptions and attitudes towards AI in healthcare, infrastructure readiness and impact of AI on clinical practice within primary care settings within PHCC.
- Is there anything that you would like to add which wasn’t discussed or covered in the FGD.
- Thank you for your participation and sharing your invaluable insight.
