# Supplementary material for: Preparing for the future: A mixed methods study protocol on AI awareness and educational integration in Qatar’s Primary Health Care Workforce
Source: PLoS One. 2026 Sep 15;21(9):e0345347. doi: 10.1371/journal.pone.0345347 (PMC13577504; doi:10.1371/journal.pone.0345347)
Supplement: Supplementary file 6 [file pone.0345347.s006.docx]

Items with different scales (binary, Likert, 0-5-10) are summed into 0-100 without justification. The 0-5-10 item disproportionately weights a single response. No reliability testing (e.g., Cronbach’s alpha) is proposed.

1. Potential systematic differences (e.g., rural population, different demographics) not addressed.
2. Target number of interviews not stated; how "past AI exposure" will be determined not specified; whether same individuals join both interviews and FGDs unclear.
3. How will Infrastructure and Human Readiness be combined (additive? weighted?)? What thresholds define "ready" vs "not ready"?
5. 4 months for transcription + thematic analysis of up to 20 interviews + 2 FGDs (10-15 each) is ambitious. Clarify resources.
6. Mixing senior and junior staff may inhibit disclosure. State whether groups will be homogeneous by role.
7. **Pilot sample (n=10) small** – Acknowledge as limitation.
8. **Reference formatting inconsistencies** – Check and correct.
9. **Text redundancy** – Abstract repeats sentences from Background. Revise.
10. **Data sharing outside PHCC** – Ethical statement should address how de-identified data will be shared with journals/reviewers.
