# Supplementary material for: Preparing for the future: A mixed methods study protocol on AI awareness and educational integration in Qatar’s Primary Health Care Workforce
Source: PLoS One. 2026 Sep 15;21(9):e0345347. doi: 10.1371/journal.pone.0345347 (PMC13577504; doi:10.1371/journal.pone.0345347)
Supplement: Supplementary file 7 [file pone.0345347.s007.docx]

Thank you for the initial assessment and check of the manuscript. The comments have been addressed as indicated below.

1. Please amend your current title to include the word "protocol" in it.

Response: Thank you for the important comment. The word protocol has been included as kindly suggested. The title reads as ‘**Preparing for the Future: A Mixed Methods Study Protocol on AI Awareness and Educational Integration in Qatar’s Primary Health Care Workforce’**

2. Please note that Study Protocols must relate to a research study that has not yet generated results. Studies that have completed either recruitment or data collection will not be considered. For further information, please see <https://journals.plos.org/plosone/s/submission-guidelines#loc-study-protocols>. Please ensure that your Methods section reports the status and timeline of the study, including an estimate of when A) participant recruitment will be completed (if applicable), B) data collection will be completed and C) results are expected. If any of these stages have already been completed, this should be indicated in the Methods section.

Response: Thank you for the important comment. The methods section includes a section on timelines of the study. The study has not been initiated yet.

**Timelines for study**

The study is tentatively expected to be initiated in April 2026. The tentative timelines for the main study tasks are summarized in the table below:

| Components of the research study | Expected completion dates (tentative) |
| --- | --- |
| Participant recruitment | April 2026- May 2026 |
| Data collection | May 2026- July 2026 |
| Drafting of results | August 2026-Nov 2026 |
