# Supplementary material for: Preparing for the future: A mixed methods study protocol on AI awareness and educational integration in Qatar’s Primary Health Care Workforce
Source: PLoS One. 2026 Sep 15;21(9):e0345347. doi: 10.1371/journal.pone.0345347 (PMC13577504; doi:10.1371/journal.pone.0345347)
Supplement: Supplementary file 8 [file pone.0345347.s008.docx]

**Response to reviewer comments**:

Dear Editor (Ahmad H. Al-Nawafleh),

We are truly grateful for the invaluable feedback provided by yourself and the reviewers. We have carefully addressed all the comments and incorporated the suggested changes.

The in-depth feedback has increased the scientific quality of the study protocol, and we are truly grateful for this.

Sincere Regards,

Dr Muslim Abbas Syed

MBChB, MPH, MS., MSc, MCPS HPE, PhD

The point-by-point responses to individual comments from reviewers 1 and 2 are provided below:

**Reviewer 1**

We thank Reviewer 1 for the positive assessment of the study rationale and for the detailed methodological recommendations. Your kind and meticulous review has further added details and strengthened the academic integrity of the protocol.

Major Comment 1

The protocol does not clearly specify the type of mixed-methods design.

**Response**

Thank you for this important observation.

We have now explicitly identified this study as a convergent mixed-methods design. Quantitative and qualitative data will be collected during the same study period, analyzed separately, and integrated during interpretation using joint displays and narrative synthesis. A dedicated subsection describing the mixed-methods design and integration procedures has been added. The relevant references have also been added as kindly advised.

Major Comment 2

There is no explanation of how quantitative and qualitative phases will be integrated.

Response

Thank you.

We have added a detailed description of integration procedures. Specifically:

- Quantitative survey findings will identify patterns, readiness levels, and educational gaps.
- Qualitative interviews and FGDs will explain and contextualize quantitative findings.
- Joint display tables will be developed to map survey findings against themes arising from qualitative analysis.
- Integration will inform the development of the proposed AI education roadmap and readiness framework.

Major Comment 3

The qualitative study design is not clearly described.

**Response**

Thank you.

We have clarified that the qualitative component will adopt a descriptive qualitative approach using semi-structured interviews and focus group discussions, with thematic analysis undertaken according to Braun and Clarke's framework.

Major Comment 4

Sampling method is not specified.

**Response**

Thank you.

The manuscript now explicitly states that maximum variation purposive sampling will be used to recruit interview and focus group participants. Variation will be sought across:

- Professional discipline
- Years of experience
- AI exposure
- Location of practice
- Clinical and administrative roles

Major Comment 5

It is unclear where interviews and FGDs will be conducted.

**Response**

Thank you.

We have added details indicating that interviews and FGDs will be conducted either:

- Face-to-face in private meeting rooms at PHCC facilities, or
- Through secure online platforms when required for operational reasons.

All sessions will occur in confidential environments.

Major Comment 6

The rationale for using both interviews and FGDs is not provided.

**Response**

Thank you.

We have expanded the qualitative methods section.

Individual interviews are intended to explore personal experiences, views, and concerns regarding AI adoption.

FGDs are intended to explore collective perspectives, group dynamics, consensus, disagreement, and shared organizational experiences that may not emerge in individual interviews.

Using both methods increases depth and triangulation.

Major Comment 7

Interview guide appears overly structured.

**Response**

Thank you.

We have revised the description of the interview guide to clarify that the questions are intended as flexible prompts rather than mandatory fixed questions. Additional probing questions will be used according to participants' responses.

The following lines are added:

The interview guide is intended to serve as a flexible framework rather than a set of mandatory, fixed questions. This approach allows participants to discuss issues that are most relevant to their experiences and perspectives while enabling the interviewer to explore emerging topics in greater depth. Additional probing questions will be used, as appropriate, to clarify responses, seek further explanation, and elicit richer insights. Such flexibility is consistent with qualitative interviewing methodology and will facilitate a more comprehensive understanding of participants’ views, experiences, and perceptions regarding the adoption and use of artificial intelligence in clinical pharmacy practice.

Major Comment 8

No explanation regarding interview guide development.

Response

Thank you.

A new section has been added describing how the interview guide was developed through:

- Literature review
- Alignment with study objectives
- Review by methodological experts

Major Comment 9

No explanation regarding pilot testing of the interview guide.

**Response**

Thank you.

We have clarified that the interview guide will undergo pilot testing with healthcare professionals who are not included in the final qualitative sample. Feedback obtained will be used to improve clarity and flow.

Major Comment 10

No explanation regarding validation of interview guide.

**Response**

Thank you.

The qualitative expert and subject matter expert reviewed the interview guide for relevance, clarity, comprehensiveness, and alignment with study objectives.

Major Comment 11

Qualitative analysis procedure is unclear.

**Response**

Thank you.

We have substantially expanded the qualitative analysis section and now describe Braun and Clarke's six-stage thematic analysis process in detail:

1. Familiarization
2. Initial coding
3. Theme generation
4. Theme review
5. Theme definition
6. Report production

Major Comment 12

Software is not specified.

**Response**

Thank you.

The revised manuscript now specifies:

- Quantitative analysis will be conducted using SPSS.
- Qualitative analysis will be supported using NVivo software.

Major Comment 13

Quantitative analysis lacks detail.

Response

Thank you.

Additional detail has been added regarding:

- Descriptive statistics
- Reliability testing
- Composite score development
- Bivariate analysis
- Multivariable regression modelling
- Missing data management

Major Comment 14

Validity process is unclear.

Response

Thank you.

We have clarified:

- The composition of the expert panel.
- Eligibility criteria for panel membership.
- Their expertise in healthcare, AI, medical education, and research methods.

Major Comment 15

Language of data collection not specified.

**Response**

Thank you.

All PHCC staff can communicate and understand English language, therefore the all data is collected in English.

Major Comment 16

Questionnaire description appears under data analysis instead of instrument section.

Response

Thank you.

The manuscript has been reorganized. Questionnaire content and domains have been moved to a dedicated "Questionnaire Development and Study Instruments" section.

Major Comment 17

FGD justification not explained.

Response

Thank you.

A dedicated justification for FGDs has been added outlining their value in identifying shared organizational perceptions and implementation challenges that may not emerge during individual interviews.

Major Comment 18

No explanation of Face Validity Index calculation.

Response

Thank you.

The validation section has been expanded to include explanation of both qualitative face validity procedures and quantitative Face Validity Index (FVI) calculations.

Major Comment 19

Several sections require additional references.

Response

Thank you.

Additional supporting literature has been incorporated throughout the revised manuscript.

**Reviewer 2**

We thank Reviewer 2 for their detailed methodological suggestions.

Comment 1

Different scale measures are combined into a 0–100 score without justification.

Response

Thank you.

We have revised the scoring methodology. Score standardization procedures and rationale are now described in detail. We have also clarified that reliability assessment, and construct evaluation will be performed before deriving composite readiness measures.

Comment 2

No reliability testing proposed.

Response

Thank you.

We have added internal consistency testing using Cronbach's alpha for multi-item scales.

Comment 3

Potential systematic population differences not addressed.

Response

Thank you.

We have added discussion on possible variation across professional groups, locations, and demographic characteristics. Stratified analyses are planned to explore these differences.

Comment 4

Target interview numbers not stated.

Response

Thank you.

The revised manuscript now states that approximately 15–20 interviews are anticipated, with final recruitment guided by thematic saturation.

Comment 5

Past AI exposure not defined.

Response

Thank you.

We have added an operational definition of AI exposure, including previous use of AI-enabled healthcare tools, formal AI education, participation in AI initiatives, or exposure to AI-supported clinical systems.

Comment 6

Whether participants can participate in both interviews and FGDs is unclear.

Response

Thank you.

We have clarified that interview and FGD participants will be recruited separately to minimize duplication and maximize diversity of perspectives.

Comment 7

How readiness scores will be combined is unclear.

Response

Thank you.

We have clarified the planned approach for integrating Infrastructure Readiness and Human Readiness measures. We have also emphasized that readiness categories are exploratory and intended primarily for framework development rather than formal diagnostic classification.

Comment 8

Timeline appears ambitious.

Response

Thank you.

We have added clarification regarding staffing, analyst involvement, and phased qualitative analysis procedures to demonstrate feasibility within the proposed timeline.

Comment 9

Mixing senior and junior staff may inhibit discussion.

Response

Thank you.

We agree. The protocol now specifies that FGDs will be organized using professionally homogeneous groups wherever possible to minimize hierarchical influences and encourage open discussion.

Comment 10

Pilot sample size is small.

Response

Thank you.

We have acknowledged this as a study limitation in the discussion section.

Comment 11

Reference formatting inconsistencies.

Response

Thank you.

All references have been reviewed and standardized according to PLOS ONE requirements.

Comment 12

Text redundancy between Abstract and Introduction.

Response

Thank you.

Redundant text has been revised and streamlined to improve readability and avoid repetition.

Comment 13

Data sharing outside PHCC should be clarified.

Response

Thank you.

The ethics and data sharing sections now specify that only fully de-identified datasets will be shared externally. No identifiable information will be released. Data sharing will comply with IRB approval, PHCC policies, and PLOS data transparency requirements.

We sincerely thank the Academic Editor and both reviewers for their valuable comments. We believe these revisions have substantially strengthened the methodological rigor, transparency, and reproducibility of the protocol.
