# Supplementary material for: Preparing for the future: A mixed methods study protocol on AI awareness and educational integration in Qatar’s Primary Health Care Workforce
Source: PLoS One. 2026 Sep 15;21(9):e0345347. doi: 10.1371/journal.pone.0345347 (PMC13577504; doi:10.1371/journal.pone.0345347)
Supplement: Peer review history

# Author comment: Author response to Decision Letter 0

**DOI:** 10.1371/journal.pone.0345347.r001

4 Mar 2026

Attachment Submitted filename: Response to reviewers.docx

# Peer review: Decision Letter 0

**DOI:** 10.1371/journal.pone.0345347.r002

**Authors:** Ahmad H. Al-Nawafleh

23 Jun 2026

Dear Dr. Syed,

Thank you for submitting your manuscript to PLOS ONE. After careful consideration, we feel that it has merit but does not fully meet PLOS ONE’s publication criteria as it currently stands. Therefore, we invite you to submit a revised version of the manuscript that addresses the points raised during the review process.

Please submit your revised manuscript by Aug 07 2026 11:59PM. If you will need more time than this to complete your revisions, please reply to this message or contact the journal office at plosone@plos.org. When you're ready to submit your revision, log on to https://www.editorialmanager.com/pone/ and select the 'Submissions Needing Revision' folder to locate your manuscript file.

- A letter that responds to each point raised by the academic editor and reviewer(s). You should upload this letter as a separate file labeled 'Response to Reviewers'.
- A marked-up copy of your manuscript that highlights changes made to the original version. You should upload this as a separate file labeled 'Revised Manuscript with Track Changes'.
- An unmarked version of your revised paper without tracked changes. You should upload this as a separate file labeled 'Manuscript'.

If you would like to make changes to your financial disclosure, please include your updated statement in your cover letter. Guidelines for resubmitting your figure files are available below the reviewer comments at the end of this letter.

If applicable, we recommend that you deposit your laboratory protocols in protocols.io to enhance the reproducibility of your results. Protocols.io assigns your protocol its own identifier (DOI) so that it can be cited independently in the future. For instructions see: https://journals.plos.org/plosone/s/submission-guidelines#loc-laboratory-protocols. Additionally, PLOS ONE offers an option for publishing peer-reviewed Lab Protocol articles, which describe protocols hosted on protocols.io. Read more information on sharing protocols at https://plos.org/protocols?utm_medium=editorial-email&utm_source=authorletters&utm_campaign=protocols.

As the corresponding author, your ORCID iD is verified in the submission system and will appear in the published article. PLOS supports the use of ORCID, and we encourage all coauthors to register for an ORCID iD and use it as well. Please encourage your coauthors to verify their ORCID iD within the submission system before final acceptance, as unverified ORCID iDs will not appear in the published article. *Only*  the individual author can complete the verification step; PLOS staff *cannot*  verify ORCID iDs on behalf of authors.

We look forward to receiving your revised manuscript.

Kind regards,

Ahmad H. Al-Nawafleh, Ph.D, MPA, CI, RN

Academic Editor

PLOS One

Journal Requirements:

When submitting your revision, we need you to address these additional requirements.

1. Please ensure that your manuscript meets PLOS ONE's style requirements, including those for file naming. The PLOS ONE style templates can be found at

https://journals.plos.org/plosone/s/file?id=wjVg/PLOSOne_formatting_sample_main_body.pdf and

https://journals.plos.org/plosone/s/file?id=ba62/PLOSOne_formatting_sample_title_authors_affiliations.pdf

2. Please provide additional details regarding participant consent. In the ethics statement in the Methods and online submission information, please ensure that you have specified (1) whether consent was informed and (2) what type you obtained (for instance, written or verbal, and if verbal, how it was documented and witnessed). If your study included minors, state whether you obtained consent from parents or guardians. If the need for consent was waived by the ethics committee, please include this information.

If you are reporting a retrospective study of medical records or archived samples, please ensure that you have discussed whether all data were fully anonymized before you accessed them and/or whether the IRB or ethics committee waived the requirement for informed consent. If patients provided informed written consent to have data from their medical records used in research, please include this information.

3.  Please include your full ethics statement in the ‘Methods’ section of your manuscript file. In your statement, please include the full name of the IRB or ethics committee who approved or waived your study, as well as whether or not you obtained informed written or verbal consent. If consent was waived for your study, please include this information in your statement as we.

4. When completing the data availability statement of the submission form, you indicated that you will make your data available on acceptance. We strongly recommend all authors decide on a data sharing plan before acceptance, as the process can be lengthy and hold up publication timelines. Please note that, though access restrictions are acceptable now, your entire data will need to be made freely accessible if your manuscript is accepted for publication. This policy applies to all data except where public deposition would breach compliance with the protocol approved by your research ethics board. If you are unable to adhere to our open data policy, please kindly revise your statement to explain your reasoning and we will seek the editor's input on an exemption. Please be assured that, once you have provided your new statement, the assessment of your exemption will not hold up the peer review process.

5. If the reviewer comments include a recommendation to cite specific previously published works, please review and evaluate these publications to determine whether they are relevant and should be cited. There is no requirement to cite these works unless the editor has indicated otherwise.

Additional Editor Comments :

Dear authors

Thank you for submitting your manuscript to Plos One.

Your protocol is very interesting and worth publication. However, it needs a major revision. Therefore I'm sending you the comments of the reviewers. Please consider every point highlighted by the reviewers and how you are handling that in a report plus the track changes document to be submitted again kind regards

[Note: HTML markup is below. Please do not edit.]

Reviewers' comments:

Reviewer's Responses to Questions

**Comments to the Author**

1. Does the manuscript provide a valid rationale for the proposed study, with clearly identified and justified research questions?

Reviewer #1: Yes

Reviewer #2: Yes

**********

2. Is the protocol technically sound and planned in a manner that will lead to a meaningful outcome and allow testing the stated hypotheses?

Reviewer #1: Partly

Reviewer #2: Partly

**********

3. Is the methodology feasible and described in sufficient detail to allow the work to be replicable?

Reviewer #1: No

Reviewer #2: Yes

**********

4. Have the authors described where all data underlying the findings will be made available when the study is complete?

The PLOS Data policy

Reviewer #1: Yes

Reviewer #2: Yes

**********

5. Is the manuscript presented in an intelligible fashion and written in standard English?

Reviewer #1: Yes

Reviewer #2: Yes

**********

Please use the space provided to explain your answers to the questions above and, if applicable, provide comments about issues authors must address before this protocol can be accepted for publication. You may also include additional comments for the author, including concerns about research or publication ethics.

You may also provide optional suggestions and comments to authors that they might find helpful in planning their study.

Reviewer #1: The proposed study addresses a highly relevant and contemporary topic, focusing on the integration of Artificial Intelligence (AI) in primary health care (PHC) settings. The topic is timely and significant, particularly given the increasing global emphasis on digital health transformation and AI-driven clinical decision-making. The study has the potential to contribute valuable insights into clinicians’ awareness, readiness, and educational needs regarding AI in PHC.

However, while the idea and research focus are strong, the methodological section requires substantial improvement, particularly the qualitative component and overall mixed-methods design.

Major Comments

Study Design and Mixed-Methods Approach

The protocol does not clearly specify the type of mixed-methods design (e.g., convergent, explanatory sequential, exploratory sequential).

There is no explanation of how the quantitative and qualitative phases will be integrated, which is a critical requirement in mixed-methods research.

The qualitative study design (e.g., phenomenology, thematic analysis, grounded theory) is not mentioned.

The qualitative component is insufficiently described and requires significant elaboration:

Sampling Strategy

The sampling method (e.g., purposive, convenience, snowball) is not specified.

Data Collection

It is unclear:

Where interviews and FGDs will be conducted

The rationale for using both interviews and focus group discussions (FGDs) is not provided.

Interview Guide

The interview guide includes 21 questions, which makes it resemble a structured questionnaire rather than a semi-structured qualitative tool.

There is no explanation of:

How the guide was developed

Whether it will be pilot tested

How it will be validated and by whom

Data Analysis Plan

The qualitative analysis section is unclear:

No description of the steps of thematic analysis is provided.

It is not mentioned whether software such as NVivo or manual coding will be used.

The quantitative analysis is also insufficiently described:

The statistical software (e.g., SPSS) is not specified.

The analysis plan lacks detail regarding statistical tests.

Validity and Reliability

Although the protocol mentions validity of the questionnaire, it is not clearly explained:

Who will assess it (experts, panel, etc.)? their credentials?

There is no mention of validation of the interview guide, which is essential for qualitative rigor.

Language Considerations

The protocol does not specify the language of data collection.

Given the study context (Qatar PHC settings), it is important to consider whether participants (e.g., administrative staff) are comfortable with English.

Translation procedures (if applicable)

Instrument Development and Structure

Details of questionnaire items are currently described in the data analysis section, which is not appropriate.

These should be moved to a dedicated section such as:

“Questionnaire Development” or

“Study Instruments”

Focus Group Discussion (FGD) Justification

The protocol does not explain:

Why FGDs are included

What additional insights they will provide beyond interviews

Missing Methodological Details

There is no explanation of how FVI (Face Validity Index) will be calculated.

Important procedural details are missing, affecting transparency and reproducibility.

Referencing

References are missing in several sections where supporting literature is expected.

This weakens the academic rigor of the protocol.

Minor Comments

Some sections require better organization and clarity.

There are minor language and grammatical issues that should be revised.

Reviewer #2: Items with different scales (binary, Likert, 0-5-10) are summed into 0-100 without justification. The 0-5-10 item disproportionately weights a single response. No reliability testing (e.g., Cronbach’s alpha) is proposed.

1. Potential systematic differences (e.g., rural population, different demographics) not addressed.

2.Target number of interviews not stated; how "past AI exposure" will be determined not specified; whether same individuals join both interviews and FGDs unclear.

3. How will Infrastructure and Human Readiness be combined (additive? weighted?)? What thresholds define "ready" vs "not ready"?

5.4 months for transcription + thematic analysis of up to 20 interviews + 2 FGDs (10-15 each) is ambitious. Clarify resources.

6. Mixing senior and junior staff may inhibit disclosure. State whether groups will be homogeneous by role.

7.Pilot sample (n=10) small – Acknowledge as limitation.

8.Reference formatting inconsistencies – Check and correct.

9.Text redundancy – Abstract repeats sentences from Background. Revise.

10.Data sharing outside PHCC – Ethical statement should address how de-identified data will be shared with journals/reviewers.

**********

what does this mean?). If published, this will include your full peer review and any attached files.

If you choose “no”, your identity will remain anonymous but your review may still be made public.

**Do you want your identity to be public for this peer review?**  For information about this choice, including consent withdrawal, please see our Privacy Policy

Reviewer #1: **Yes:**  Wajiha Rehman

Reviewer #2: No

**********

[NOTE: If reviewer comments were submitted as an attachment file, they will be attached to this email and accessible via the submission site. Please log into your account, locate the manuscript record, and check for the action link "View Attachments". If this link does not appear, there are no attachment files.]

To ensure your figures meet our technical requirements, please review our figure guidelines: https://journals.plos.org/plosone/s/figures

You may also use PLOS’s free figure tool, NAAS, to help you prepare publication quality figures: https://journals.plos.org/plosone/s/figures#loc-tools-for-figure-preparation.

NAAS will assess whether your figures meet our technical requirements by comparing each figure against our figure specifications.

Attachment Submitted filename: Items with different scales.docx

# Author comment: Author response to Decision Letter 1

**DOI:** 10.1371/journal.pone.0345347.r003

3 Aug 2026

Response to reviewer comments:

Dear Editor (Ahmad H. Al-Nawafleh),

We are truly grateful for the invaluable feedback provided by yourself and the reviewers. We have carefully addressed all the comments and incorporated the suggested changes.

The in-depth feedback has increased the scientific quality of the study protocol, and we are truly grateful for this.

Sincere Regards,

Dr Muslim Abbas Syed

MBChB, MPH, MS., MSc, MCPS HPE, PhD

The point-by-point responses to individual comments from reviewers 1 and 2 are provided below:

Reviewer 1

We thank Reviewer 1 for the positive assessment of the study rationale and for the detailed methodological recommendations. Your kind and meticulous review has further added details and strengthened the academic integrity of the protocol.

Major Comment 1

The protocol does not clearly specify the type of mixed-methods design.

Response

Thank you for this important observation.

We have now explicitly identified this study as a convergent mixed-methods design. Quantitative and qualitative data will be collected during the same study period, analyzed separately, and integrated during interpretation using joint displays and narrative synthesis. A dedicated subsection describing the mixed-methods design and integration procedures has been added. The relevant references have also been added as kindly advised.

Major Comment 2

There is no explanation of how quantitative and qualitative phases will be integrated.

Response

Thank you.

We have added a detailed description of integration procedures. Specifically:

• Quantitative survey findings will identify patterns, readiness levels, and educational gaps.

• Qualitative interviews and FGDs will explain and contextualize quantitative findings.

• Joint display tables will be developed to map survey findings against themes arising from qualitative analysis.

• Integration will inform the development of the proposed AI education roadmap and readiness framework.

Major Comment 3

The qualitative study design is not clearly described.

Response

Thank you.

We have clarified that the qualitative component will adopt a descriptive qualitative approach using semi-structured interviews and focus group discussions, with thematic analysis undertaken according to Braun and Clarke's framework.

Major Comment 4

Sampling method is not specified.

Response

Thank you.

The manuscript now explicitly states that maximum variation purposive sampling will be used to recruit interview and focus group participants. Variation will be sought across:

• Professional discipline

• Years of experience

• AI exposure

• Location of practice

• Clinical and administrative roles

Major Comment 5

It is unclear where interviews and FGDs will be conducted.

Response

Thank you.

We have added details indicating that interviews and FGDs will be conducted either:

• Face-to-face in private meeting rooms at PHCC facilities, or

• Through secure online platforms when required for operational reasons.

All sessions will occur in confidential environments.

Major Comment 6

The rationale for using both interviews and FGDs is not provided.

Response

Thank you.

We have expanded the qualitative methods section.

Individual interviews are intended to explore personal experiences, views, and concerns regarding AI adoption.

FGDs are intended to explore collective perspectives, group dynamics, consensus, disagreement, and shared organizational experiences that may not emerge in individual interviews.

Using both methods increases depth and triangulation.

Major Comment 7

Interview guide appears overly structured.

Response

Thank you.

We have revised the description of the interview guide to clarify that the questions are intended as flexible prompts rather than mandatory fixed questions. Additional probing questions will be used according to participants' responses.

The following lines are added:

The interview guide is intended to serve as a flexible framework rather than a set of mandatory, fixed questions. This approach allows participants to discuss issues that are most relevant to their experiences and perspectives while enabling the interviewer to explore emerging topics in greater depth. Additional probing questions will be used, as appropriate, to clarify responses, seek further explanation, and elicit richer insights. Such flexibility is consistent with qualitative interviewing methodology and will facilitate a more comprehensive understanding of participants’ views, experiences, and perceptions regarding the adoption and use of artificial intelligence in clinical pharmacy practice.

Major Comment 8

No explanation regarding interview guide development.

Response

Thank you.

A new section has been added describing how the interview guide was developed through:

• Literature review

• Alignment with study objectives

• Review by methodological experts

Major Comment 9

No explanation regarding pilot testing of the interview guide.

Response

Thank you.

We have clarified that the interview guide will undergo pilot testing with healthcare professionals who are not included in the final qualitative sample. Feedback obtained will be used to improve clarity and flow.

Major Comment 10

No explanation regarding validation of interview guide.

Response

Thank you.

The qualitative expert and subject matter expert reviewed the interview guide for relevance, clarity, comprehensiveness, and alignment with study objectives.

Major Comment 11

Qualitative analysis procedure is unclear.

Response

Thank you.

We have substantially expanded the qualitative analysis section and now describe Braun and Clarke's six-stage thematic analysis process in detail:

1. Familiarization

2. Initial coding

3. Theme generation

4. Theme review

5. Theme definition

6. Report production

Major Comment 12

Software is not specified.

Response

Thank you.

The revised manuscript now specifies:

• Quantitative analysis will be conducted using SPSS.

• Qualitative analysis will be supported using NVivo software.

Major Comment 13

Quantitative analysis lacks detail.

Response

Thank you.

Additional detail has been added regarding:

• Descriptive statistics

• Reliability testing

• Composite score development

• Bivariate analysis

• Multivariable regression modelling

• Missing data management

Major Comment 14

Validity process is unclear.

Response

Thank you.

We have clarified:

• The composition of the expert panel.

• Eligibility criteria for panel membership.

• Their expertise in healthcare, AI, medical education, and research methods.

Major Comment 15

Language of data collection not specified.

Response

Thank you.

All PHCC staff can communicate and understand English language, therefore the all data is collected in English.

Major Comment 16

Questionnaire description appears under data analysis instead of instrument section.

Response

Thank you.

The manuscript has been reorganized. Questionnaire content and domains have been moved to a dedicated "Questionnaire Development and Study Instruments" section.

Major Comment 17

FGD justification not explained.

Response

Thank you.

A dedicated justification for FGDs has been added outlining their value in identifying shared organizational perceptions and implementation challenges that may not emerge during individual interviews.

Major Comment 18

No explanation of Face Validity Index calculation.

Response

Thank you.

We did not calculate a Face Validity Index (FVI) because face validity is inherently a subjective assessment rather than a psychometric property requiring formal quantification. Instead, face validity will be assessed qualitatively through pilot testing with prospective participants, while formal instrument evaluation will be conducted using expert review and Content Validity Ratio (CVR) analysis.

Major Comment 19

Several sections require additional references.

Response

Thank you.

Additional supporting literature has been incorporated throughout the revised manuscript.

Reviewer 2

We thank Reviewer 2 for their detailed methodological suggestions.

Comment 1

Different scale measures are combined into a 0–100 score without justification.

Response

Thank you.

We have revised the scoring methodology. Score standardization procedures and rationale are now described in detail. We have also clarified that reliability assessment, and construct evaluation will be performed before deriving composite readiness measures.

Comment 2

No reliability testing proposed.

Response

Thank you.

We have added internal consistency testing using Cronbach's alpha for multi-item scales.

Comment 3

Potential systematic population differences not addressed.

Response

Thank you.

We have added discussion on possible variation across professional groups, locations, and demographic characteristics. Stratified analyses are planned to explore these differences.

Comment 4

Target interview numbers not stated.

Response

Thank you.

The revised manuscript now states that approximately 15–20 interviews are anticipated, with final recruitment guided by thematic saturation.

Comment 5

Past AI exposure not defined.

Response

Thank you.

We have added an operational definition of AI exposure, including previous use of AI-enabled healthcare tools, formal AI education, participation in AI initiatives, or exposure to AI-supported clinical systems.

Comment 6

Whether participants can participate in both interviews and FGDs is unclear.

Response

Thank you.

We have clarified that interview and FGD participants will be recruited separately to minimize duplication and maximize diversity of perspectives.

Comment 7

How readiness scores will be combined is unclear.

Response

Thank you.

We have clarified the planned approach for integrating Infrastructure Readiness and Human Readiness measures. We have also emphasized that readiness categories are exploratory and intended primarily for framework development rather than formal diagnostic classification.

Comment 8

Timeline appears ambitious.

Response

Thank you.

We have added clarification regarding staffing, analyst involvement, and phased qualitative analysis procedures to demonstrate feasibility within the proposed timeline.

Comment 9

Mixing senior and junior staff may inhibit discussion.

Response

Thank you.

We agree. The protocol now specifies that FGDs will be organized using professionally homogeneous groups wherever possible to minimize hierarchical influences and encourage open discussion.

Comment 10

Pilot sample size is small.

Response

Thank you.

We have acknowledged this as a study limitation in the discussion section.

Comment 11

Reference formatting inconsistencies.

Response

Thank you.

All references have been reviewed and standardized according to PLOS ONE requirements.

Comment 12

Text redundancy between Abstract and Introduction.

Response

Thank you.

Redundant text has been revised and streamlined to improve readability and avoid repetition.

Comment 13

Data sharing outside PHCC should be clarified.

Response

Thank you.

The ethics and data sharing sections now specify that only fully de-identified datasets will be shared externally. No identifiable information will be released. Data sharing will comply with IRB approval, PHCC policies, and PLOS data transparency requirements.

We sincerely thank the Academic Editor and both reviewers for their valuable comments. We believe these revisions have substantially strengthened the methodological rigor, transparency, and reproducibility of the protocol.

Attachment Submitted filename: response to reviewer comments.docx

# Editor report: Decision Letter 1

**DOI:** 10.1371/journal.pone.0345347.r004

**Authors:** Ahmad H. Al-Nawafleh

17 Aug 2026

Preparing for the Future: A Mixed Methods Study Protocol on AI Awareness and Educational Integration in Qatar’s Primary Health Care Workforce.

PONE-D-26-10681R1

Dear Dr. Syed,

We’re pleased to inform you that your manuscript has been judged scientifically suitable for publication and will be formally accepted for publication once it meets all outstanding technical requirements.

Within one week, you’ll receive an e-mail detailing the required amendments. When these have been addressed, you’ll receive a formal acceptance letter and your manuscript will be scheduled for publication.

An invoice will be generated when your article is formally accepted. Please note, if your institution has a publishing partnership with PLOS and your article meets the relevant criteria, all or part of your publication costs will be covered. Please make sure your user information is up-to-date by logging into Editorial Manager at Editorial Manager® and clicking the ‘Update My Information' link at the top of the page. For questions related to billing, please contact billing support.

If your institution or institutions have a press office, please notify them about your upcoming paper to help maximize its impact. If they’ll be preparing press materials, please inform our press team as soon as possible -- no later than 48 hours after receiving the formal acceptance. Your manuscript will remain under strict press embargo until 2 pm Eastern Time on the date of publication. For more information, please contact onepress@plos.org.

Kind regards,

Ahmad H. Al-Nawafleh, Ph.D, MPA, CI, RN

Academic Editor

PLOS One

Additional Editor Comments (optional):

Reviewers' comments:

# Editor report: Acceptance letter

**DOI:** 10.1371/journal.pone.0345347.r005

**Authors:** Ahmad H. Al-Nawafleh

PONE-D-26-10681R1

PLOS One

Dear Dr. Syed,

I'm pleased to inform you that your manuscript has been deemed suitable for publication in PLOS One. Congratulations! Your manuscript is now being handed over to our production team.

At this stage, our production department will prepare your paper for publication. This includes ensuring the following:

* All references, tables, and figures are properly cited

* All relevant supporting information is included in the manuscript submission,

* There are no issues that prevent the paper from being properly typeset

You will receive further instructions from the production team, including instructions on how to review your proof when it is ready. Please keep in mind that we are working through a large volume of accepted articles, so please give us a few days to review your paper and let you know the next and final steps.

Lastly, if your institution or institutions have a press office, please let them know about your upcoming paper now to help maximize its impact. If they'll be preparing press materials, please inform our press team within the next 48 hours. Your manuscript will remain under strict press embargo until 2 pm Eastern Time on the date of publication. For more information, please contact onepress@plos.org.

You will receive an invoice from PLOS for your publication fee after your manuscript has reached the completed accept phase. If you receive an email requesting payment before acceptance or for any other service, this may be a phishing scheme. Learn how to identify phishing emails and protect your accounts at https://explore.plos.org/phishing.

If we can help with anything else, please email us at customercare@plos.org.

Thank you for submitting your work to PLOS One and supporting open access.

Kind regards,

PLOS One Editorial Office Staff

on behalf of

Prof. Ahmad H. Al-Nawafleh

Academic Editor

PLOS One
